# Deep learning-based noise filtering toward millisecond order imaging by using scanning transmission electron microscopy

**DOI:** 10.1038/s41598-022-17360-3

**Published:** 2022-08-05

**Authors:** Shiro Ihara, Hikaru Saito, Mizumo Yoshinaga, Lavakumar Avala, Mitsuhiro Murayama

**Affiliations:** 1 Institute for Materials Chemistry and Engineering, Kyushu University, Fukuoka, 816-8580 Japan; 2 Interdisciplinary Graduate School of Engineering Sciences, Kyushu University, Fukuoka, 816-8580 Japan; 3 Pan-Omics Data-Driven Research Innovation Center, Kyushu University, Fukuoka, 816-8580 Japan; 4 Department of Materials Science and Engineering, Virginia Tech, Blacksburg, VA 24061 USA; 5 Reactor Materials and Mechanical Design Group, Energy and Environmental Directorate, Pacific Northwest National Laboratory, Richland, WA 99354 USA

**Keywords:** Microscopy, Transmission electron microscopy

## Abstract

Application of scanning transmission electron microscopy (STEM) to in situ observation will be essential in the current and emerging data-driven materials science by taking STEM’s high affinity with various analytical options into account. As is well known, STEM’s image acquisition time needs to be further shortened to capture a targeted phenomenon in real-time as STEM’s current temporal resolution is far below the conventional TEM’s. However, rapid image acquisition in the millisecond per frame or faster generally causes image distortion, poor electron signals, and unidirectional blurring, which are obstacles for realizing video-rate STEM observation. Here we show an image correction framework integrating deep learning (DL)-based denoising and image distortion correction schemes optimized for STEM rapid image acquisition. By comparing a series of distortion corrected rapid scan images with corresponding regular scan speed images, the trained DL network is shown to remove not only the statistical noise but also the unidirectional blurring. This result demonstrates that rapid as well as high-quality image acquisition by STEM without hardware modification can be established by the DL. The DL-based noise filter could be applied to in-situ observation, such as dislocation activities under external stimuli, with high spatio-temporal resolution.

## introduction

Transmission electron microscopy (TEM) is commonly used in various fields such as materials science^1,2^, biology^3^, and so on. Recent advancement in TEM technique has enabled us to conduct various kinds of in situ observation with different external stimuli, e.g., loading^4,5^, heating^6,7^, etc. In-situ observation in TEM can capture real-time temporal evolution of materials even at the atomic scale. Meanwhile, probing the temporal evolution of materials requires additional considerations. Currently, recent developments in imaging devices have allowed conventional TEM (CTEM) is the primary candidate for in situ observation. However, the resulting data fidelity is sensitive to the sample thickness in the CTEM mode due to the chromatic aberration of the objective lenses; the sample thickness must typically be thinner than 100 nm for high resolution imaging. Multiple factors, such avoiding chromatic aberration^8,9^ in the imaging lenses and somewhat improved robustness to dynamical diffraction effects, mean that Scanning TEM (STEM) is the technique of choice for high-resolution analytical studies of crystalline materials. The STEM can visualize even thicker samples, such as three-dimensional structures of dislocations in a steel with a thickness of 300 nm or more^10,11^, micro-meter dimensions of polymer samples^12^ and biological cells with a thickness of 1 $\upmu$m^9^. The working principle of the STEM that the targeted area is scanned and imaged point-by-point by a converged electron beam leads a lower temporal resolution in principle compared to the CTEM using a continuous illumination. This limitation in temporal resolution makes the STEM difficult to be deployed as is.

It should be noted that the current limitation in temporal resolution is not from the scanning speed itself. The commercial STEM has already realized the scanning speed of 100 ns/pixel or faster^13^, which offers several tens of millisecond per frame for an image size of 512 × 512 pixels, reaching a video-rate. Such a rapid scanning, however, brings about poor signal intensity and artifacts. The former originates from much shorter dwell time per pixel and the latter, i.e., unidirectional blurring and image distortion in the scanning direction, mainly comes from the hardware working principles. The poor signal intensity causes a non-negligible statistical noise which is also well recognized in the rapid image acquisition by using the CTEM^14,15^. The unidirectional blurring and distortion, on the other hand, are unique in the STEM and originated from the delay of the detector response as well as the hysteresis effect of the scanning device^16^. Since the scanning speed and the image quality are basically in a trade-off relationship, high quality as well as high-speed image acquisition would be challenging without developing a creative approach. This problem is worth conquering for establishing a STEM-based in-situ observation framework particularly for meso-scale phenomena.

As the first step in the framework development, we have proposed a deep learning (DL)-based noise filter^16^. Although DL application to image processing and analysis mostly focus on detection^17,18^, segmentation^19^, and classification^20^ of objects, DL is also utilized as a denoising technique^21^ in the field of electron microscopy. The advantage of the DL-based noise filter is that the noise filter does not have to assume noise types. The vast majority of noise filters proposed so far limitedly work for noises with predictable analytical expressions such as additive^22,23^, multiplicative^24^, Poisson^25,26^, and high frequency^27^ types. In a similar case to the STEM, in an atomic force microscope (AFM), where a cantilever scans a sample surface, stripe noise emerges when sample surface height rapidly changes. In this case, an FFT-based denoising scheme has been proposed^28^. The DL-based noise filtering has also been reported for AFM^29^, where the DL-based approach showed robust performance for removing both periodic and non-periodic stripes. On the other hand, the artifacts arising in the rapid STEM imaging are complicatedly combined with statistical noises, and the resultant artifacts or noises are difficult to predict in advance. Our previous study^16^ has pointed out that the BM3D (block matching 3D)^30,31^, which is well known to be highly effective for removing the statistical noise, could not satisfactorily work for rapid scan STEM image denoising. In this particular study, therefore, a DL-based noise filter was developed for the rapid STEM imaging, leading to considerable improvement of speed and accuracy in STEM tomography^16^.

Based on our initial success, this study aims to develop further to improve the performance and versatility of DL-based noise filtering technique. Here, STEM images of dislocations are used as a model case. The DL approach performed in this paper demonstrates that it successfully removes not only the statistical noise but also the unidirectional blurring, which could not be achieved by the DL approach proposed in the previous study^16^. The training is not a same way as in the previous study because of the image distortion. Since the training without distortion correction risks artifact generation, it must be corrected prior to a training. Although STEM image distortion correction algorithms have been proposed so far, the vast majority of their applications are atomic resolution images, where periodicity of an image can be assumed^32,33^ and a share deformation of image due to sample drift is targeted^34,35^. Therefore, the distortion correction method presented in this study is developed at first. In this paper, we firstly describe the concept of the denoising approach, where a summary of STEM image properties and the procedure of the DL are discussed. The distortion correction algorithm is also briefly described. Then, the performance of the newly designed DL filtering is demonstrated according to the resulting data fidelity. As noted in^36,37^, diffraction contrast imaging STEM , which utilizes both bright field (BF) and annular dark field (ADF) detectors, has demonstrated that this method is able to obtain quantitative information from a thicker sample region (foil thickness > 200 nm) than CTEM approach by removing undesired diffraction contrast elements such as bend contour. Thus, the present technical development would add the STEM further potential to be applied to data driving electron microscopy. The denoising technique proposed in this study would be a standard tool for rapid image acquisition by the STEM, which would be applicable to in-situ observations, including but not limited to dislocation ensembles under external stress or heating.

## Concept of STEM image processing

**Figure 1:**
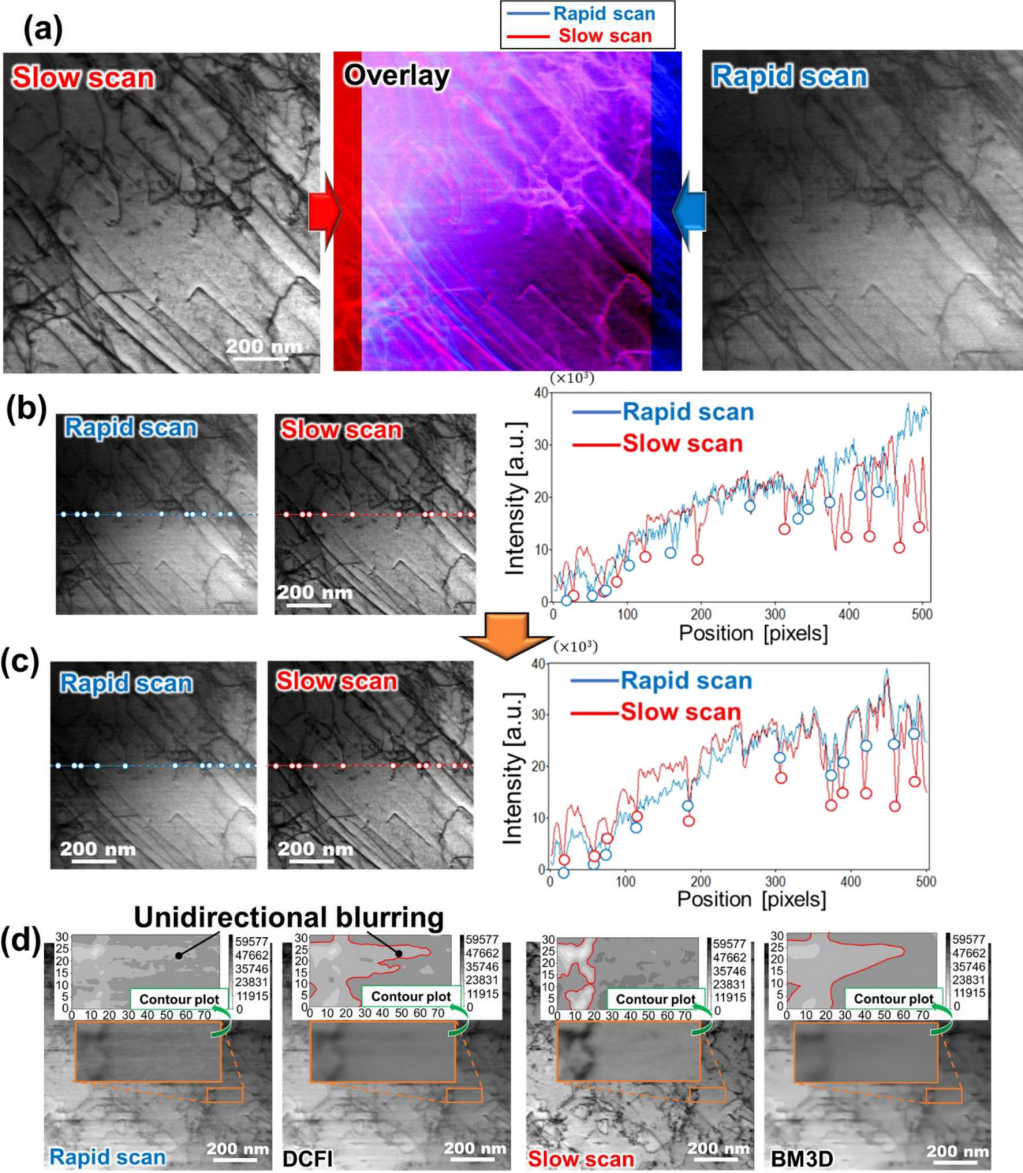
STEM images obtained by different acquisition speed and those properties. Rapid scan of 100 ns/pixel causes image distortion as clearly represented by (**a**) superposition with STEM image acquired by slow scan of 5 $\upmu$s/pixel. The distortion is to be corrected by the way described in "Method" section. Line profiles (**b**) before and (**c**) after distortion correction show the discrepancy at dips representing dislocations is modified by the distortion correction. (**d**) The rapid scanning brings about poor electron signals and delay of detector response, causing statistical noise and unidirectional image blurring, respectively. Although the drift-corrected-frame-integration (DCFI), which is made by averaging 50 frames of rapid scan images, reduces the statistical noise, thus it shows rather smooth contour plot, the blurring remains. The blurring also remains even if the image is processed by well-known noise filter, block-matching-and-three-dimensional (BM3D)^31^, while the slow scan image contains no blurring. Note that the FOV shown in (**d**) were distortion corrected images and not used during training process.

A STEM image of dislocations obtained by a rapid scanning, 100 ns/pixel, exhibits image distortion. The distortion is clearly visible in Fig. 1a where an image obtained by slow scanning, 5 $\upmu$s/pixel, is superposed for comparison. The viewing area of the rapid scan image was broadened and distorted non-linearly compared to the slow scan image. Since distortion is likely caused by hysteresis of the electron beam scanning coils, this problem is inevitable in regular commercial electron microscopes. It should be noted that we have acquired the series of images after confirming that the sample was not drifting. Thus, the distortion that originates from sample drift was negligible in this study. It was also confirmed that the slow scanning did not cause image distortion as represented in Supplementary information A. Therefore, the distortion which is caused by the scanning device is considered in this study.

The image distortion makes the training process inefficient or impossible at worst as shown in Supplementary information B, where the DL filter trained without distortion correction was applied to validation images, because a DL network will be forced to recognize the distortion and other artifacts including statistical noise at the same time (the training condition was same as the following training). Thus, the image distortion must be corrected before the training. In the distortion correction process, a local image distortion in a certain part of a frame was calculated by finding the optimal linear transformation scoring the highest cross-correlation between a rapid scan image and the corresponding slow scan image. Then, this process was successively performed over the whole of the frames. Note that the image distortion was reasonably assumed to be a linear within a small width parallel to the scan direction. Technical details of the distortion correction are described in the "Method" section. Figures 1b and c show line profiles extracted from a rapid scan image and a slow scan image, before and after the distortion correction, respectively. In the figures, discrepancies in dip positions, which correspond to the location of dislocations marked by the solid circles (Fig. 1b), were recognized and then corrected by the distortion correction (Fig. 1c).

The rapid scanning also introduces the statistical noise and unidirectional blurring originating from low electron counts, i.e., poor signal-to-noise ratio, and the delay of the detector response, respectively. Figure 1d compares the rapid scan image and the drift-corrected-frame-integration (DCFI) image, which is created by averaging 50 frames of the rapid scan image obtained from the same field-of-view (FOV) by using the commercial software, Velox™ (Thermo Fisher Scientific Inc.). Although the DCFI creation algorithm is proprietary, it appears to involve a sum of individual frames aligned by cross-correlation^38^. For the sake of visibility, the contour plot of a magnified view is also shown in Fig. 1d. Note that the FOV shown in the figure were distortion corrected images and not used during training process. In Fig. 1d, the rapid scan image gives rather rough contour curve while the DCFI’s contour curve appears much smoother. The statistical noise caused the roughness in the rapid scan image and the noise was removed by the DCFI’s averaging process. The unidirectional blurring generated by the rapid scanning, however, was not removed in the DCFI image as clearly seen in the contour plot. Since the slow scan image in Fig. 1d exhibits no blurring, the blurring is a rapid scanning-specific artifact. Even if the DCFI process improved the signal intensity by averaging 50 frames of rapid scan image, the unidirectional blurring remained. Therefore, a noise filter must be developed and deployed to eliminate the rapid scanning-specific artifact. However, as shown in Fig. 1d bottom-right, a conventional noise filter, for example, the block-matching-and-three-dimensional (BM3D)^31^, could not eliminate the unidirectional blurring, although the parameter of noise type and its variance were optimized so that the peak-signal-to-noise-ratio (PSNR), which is defined in the "Method" section, between the filtered rapid scan image and the slow scan image scored the highest. To eliminate undesirable objects from an image by using a non-learning noise filter, in general, the mathematical expression of the targets has to be defined a priori. To our knowledge, the rapid scanning-specific-type artifact is not formulated so far. A DL approach should be capable of learning and correcting rapid scanning noise given sufficient training data with distortion corrected rapid scan images and slow scan images. Once trained the network can be applied to rapid scan images without the necessity of recording slow-scan images. In this paper, we demonstrate noise reduction by training a distortion corrected dataset of 50 frames of 50 different FOVs with corresponding slow scan images.

It is worth noting that the shape of the unidirectional blurring might depend on the signal intensity of dislocations or the shape of those as represented in Fig. 1d most-left. In the upper part of the contour plot, where the dislocation with a relatively higher signal intensity lies horizontally, which is the scanning direction of the STEM, the unidirectional blurring prolonged compared to the lower part of the plot. This shape or intensity dependency makes the modeling of the unidirectional blurring more challenging.

**Figure 2:**
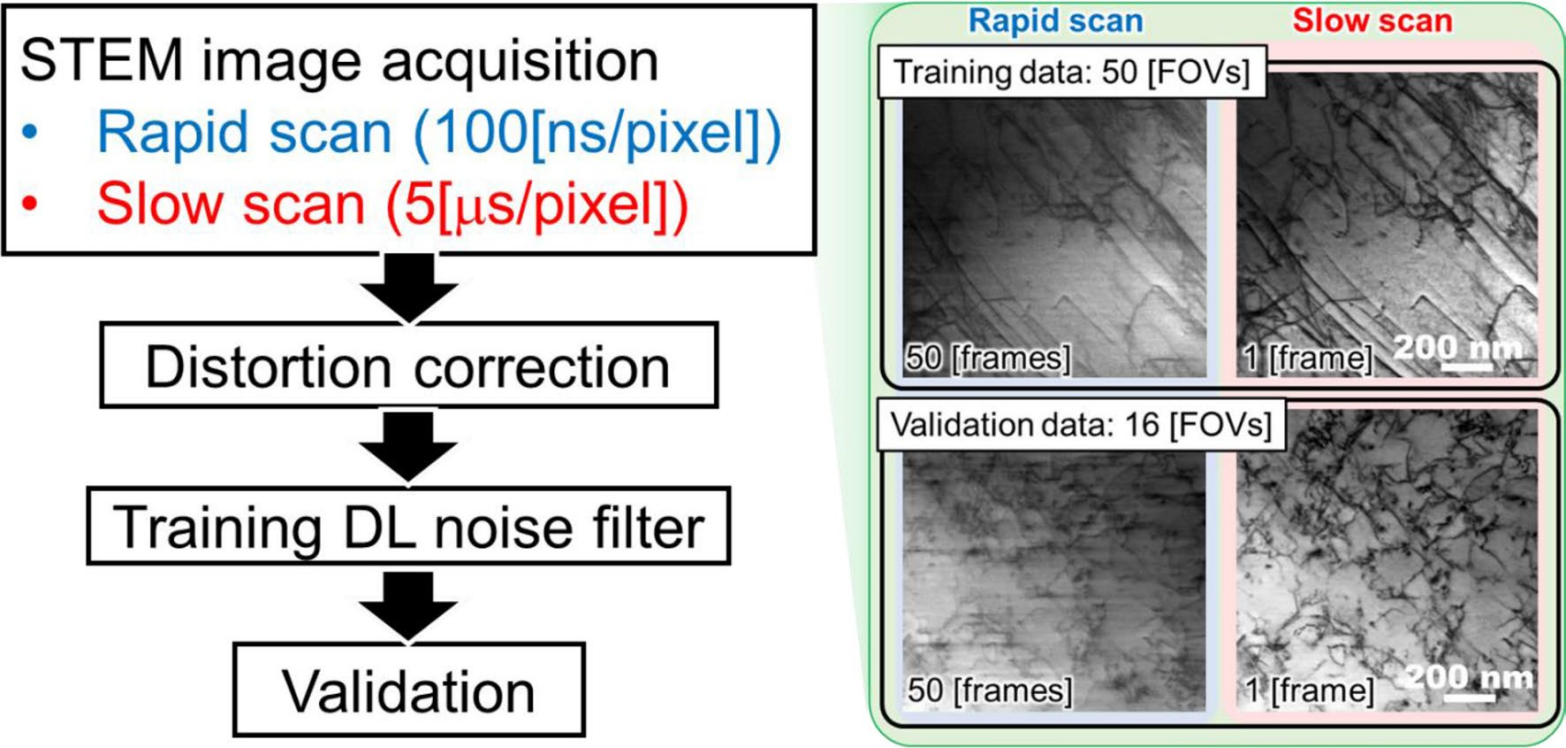
Flow chart of this study. Example images of a training dataset and a validation dataset are also shown.

The basic idea of the use of DL is that the DL network could be capable of denoising the unknown and/or not-formulated type artifacts as long as sufficient number of training data sets would be obtained. The previous study has demonstrated that the adequately trained DL-based noise filter showed better denoising performance than the BM3D^16^. The unidirectional blurring, however, could not be completely eliminated because the DCFI images were used as the reference. Although the training between the rapid scan images and the DCFI images does not necessitate the distortion correction, thus making the necessary training setup straightforward, there is room for improvement in denoising performance. Since the slow scan images are free from the unidirectional blurring, the denoising performance could further be improved by using those as the training dataset. For summary, the flowchart of this study is indicated in Fig. 2. A set of distortion corrected 50 frames × 50 FOVs rapid scan images and the corresponding slow scan images were used for a training of U-net^39^ as a proof-of-concept attempt. The details of the distortion correction process and the training are described in the "Method" section. Then, 50 frames × 16 FOVs of validation images were processed through the trained DL filter.

## Results

### Performance of the DL filter

**Figure 3:**
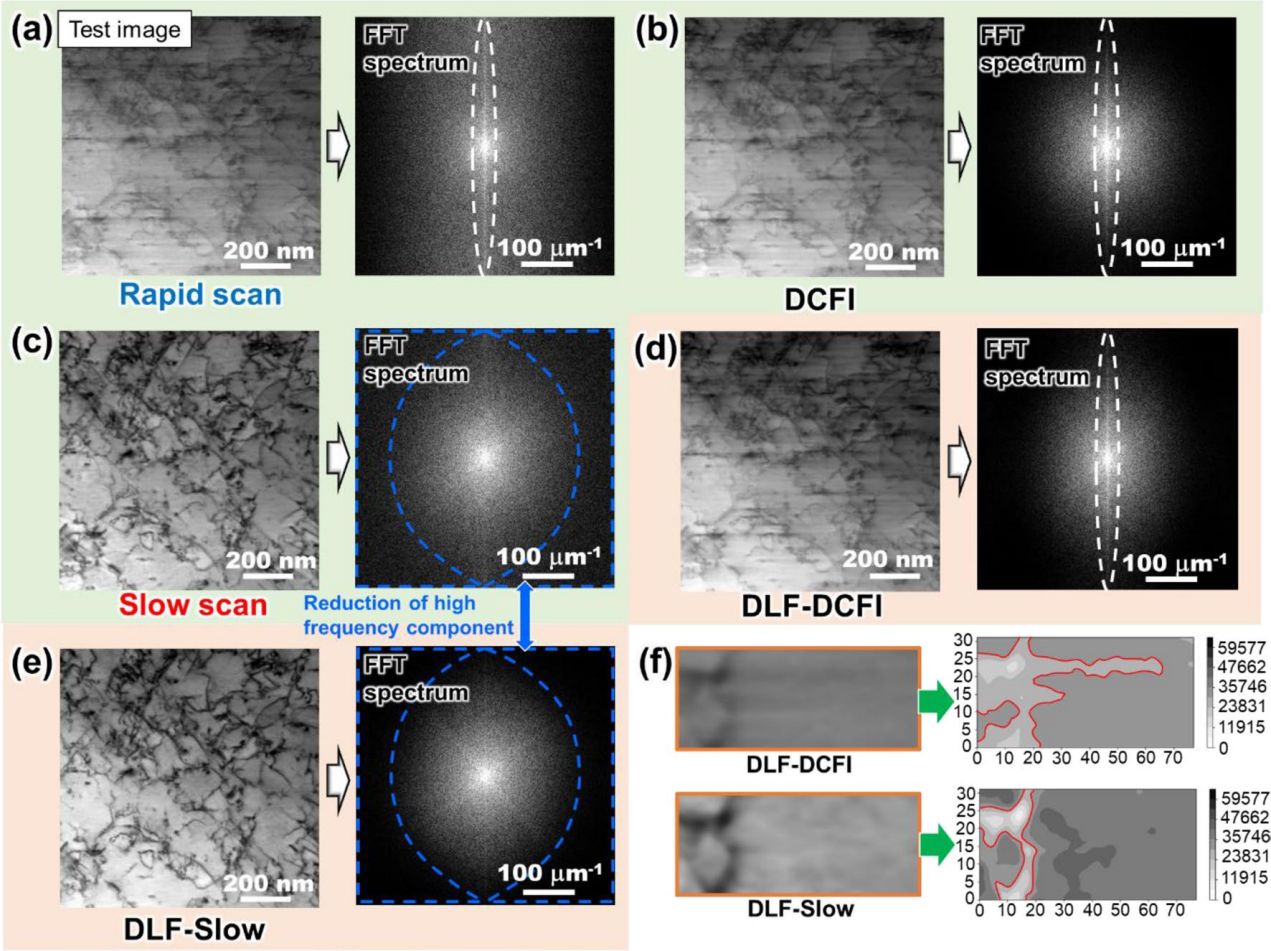
Sample STEM images selected from a validation dataset of 50 frames × 16 FOVs. The rest of results are displayed in Supplementary information B. (**a**) Rapid scan, (**b**) DCFI, (**c**) slow scan, DL filtered rapid scan supervised by (**d**) DCFI images (DLF-DCFI), and (**e**) slow scan images (DLF-Slow) are represented. This figure also shows FFT spectra of those images, where the vertical pattern in FFT indicating the unidirectional blurring is circled by broken line. (**f**) Contour plot of the partial DLF-DCFI image demonstrates that the blurring remains, while the DLF-Slow completely removes it. Note that all the FFT spectrum images displayed in this paper are obtained by using DigitalMicrograph (Gatan, Inc.) after applying the Hanning window.

A distortion corrected image dataset of 50 frames × 16 FOVs was denoised through the DL filter trained by the slow scan images. Also, the result of the DCFI image-trained DL filter is shown in this section for comparison. Figures 3a–c show a representative rapid scan image in the validation image dataset and its two corresponding references, i.e., the DCFI image and the slow scan image, respectively. The original image set (Fig. 3a) was denoised through the DL filter trained by using the DCFI image set (Fig. 3d DLF-DCFI) or the slow scan image set (Fig. 3e DLF-Slow). The results of identical denoising for other FOVs are shown in Supplementary information B. In Figs. 3d and e, the DLF-DCFI remained blurred while the statistical noise and the unidirectional blurring were successfully removed in the DLF-Slow, as clearly represented in the contour plots match-up (Fig. 3f). This is expectable because the DCFI images originally contained the blur. As noted in the previous section, the advantage of the use of the DCFI images as a reference data is that since there is no distortion between the rapid images and the DCFI images, the DL network can be trained without the distortion correction. Because the distortion correction developed in this study requires calculating local image cross-correlation between the deformed rapid scan image and the corresponding part of slow scan image, distortion correction of images with too poor electron signal or those containing a significant amount of noise would be challenging. The DLF-DCFI might be required for denoising such images at the expense of removing the rapid scanning-specific artifacts. The superior performance of DLF-Slow is demonstrated by the comparison of FFT patterns from these images, where a vertical streak (circled by broken lines) in the original image was removed in the DLF-Slow unlike in the DLF-DCFI. Since the direction of FFT spectra appears perpendicular to the lines in the original image, the unidirectional blurring, which extends in the horizontal direction (the scanning direction of the STEM), has appeared as an intense vertical feature in the FFT of Figs. 3a,b and d. Again, the DLF-Slow has the least vertical feature as well as the least high frequency component indicating the statistical noise. The removal of the statistical noise by DLF-Slow is not trivial since the slow scan images, which were used as the reference images in the training process, contained the high frequency component as marked by the broken line in Fig. 3c. Actually, the statistical noise seems to be more or less evenly distributed in slow scan images, and the noise might be reduced through the DLF-Slow, suggesting that output of the DLF-Slow could be even more noise-free than the reference images. Likely this is because the DLF was tuned so that the mean squared error between “all” the output images and the reference images was minimized, i.e., common features of the reference images selectively survive through the filtering process, resulting in the elimination of random components such as the statistical noise.

**Figure 4:**
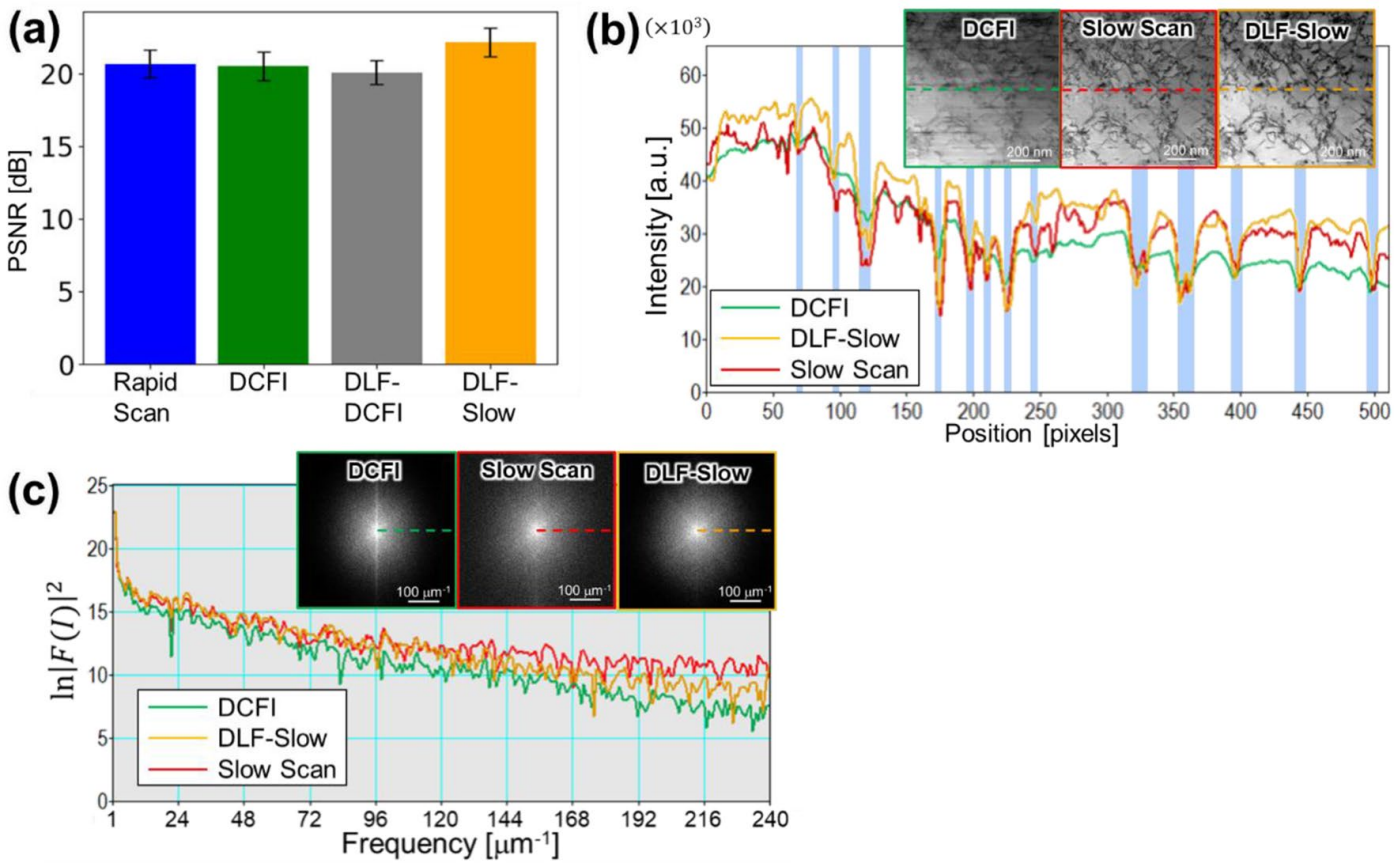
Evaluations of DL filtered images. (**a**) DLF-Slow images show highest PSNR to slow scan images among others, with about 7% increase in average from original rapid scan images. The little increase comes from the difference of back ground intensity as represented in (**b**) line profiles of each image. In the figure, it is demonstrated that the DLF-Slow mainly matches the intensity at the dislocation dips marked by light-blue stripes. (**c**) Line profiles of FFT spectrum image, which shows natural logarithm of power spectrum of FFT processed image *F*(*I*), represents good agreement between the slow scan image and the DLF-Slow image within the low frequency region up to 120 $\upmu$m^−1^, while the line profile of FFT spectrum in the DCFI image is little agreement with that in the slow scan image.

We evaluated the PSNR, which is a typical parameter representing an image quality, relative to the slow scan images for the rapid scan, the DCFI, the DLF-DCFI, and the DLF-Slow images. Here, the signal intensity for each image was scaled so that the minimum and the maximum are 0 and 65,535, respectively, prior to the calculation. Figure 4a shows the average of PSNR for all four types of images with an error bar indicating the standard deviation of the PSNR is given. The figure represents that the DLF-Slow images showed the highest PSNR among the four, increased by about 7% from the original images on average. Although the noise was successfully removed by the DLF-Slow operation as shown in Fig. 3e, the PSNR was not largely increased so much. This is because of the background intensity difference between the slow scan images and the DLF-Slow images as the line profiles of two images demonstrated in Fig. 4b. The signal intensity of the DLF-Slow image (orange) matched with that of the slow scan image (red) at nearly all dips (light-blue highlighted) that represent the dislocations. At the rest of locations, however, two profiles were not well aligned. By referring to the intensity profile of the DCFI image (green), this result would come from the fact that the background intensity of rapid scan image originally differed from that of the slow scan image. Note that in the DCFI image, the statistical noise has been reduced by averaging 50 frames of the rapid scan image, thus the profile of the DCFI image indicated the net signal obtained by the rapid scanning.

The background difference might be caused by the difference in the BF detector response between the rapid scan at 100 ns/pixel and the slow scan at 5 $\upmu$s/pixel. The detector's response could vary depending on the dose of an incident electron beam. If that is the case, the signal intensity profile of these two conditions would not be equal. To clarify the reasons why the detector response appears to be unlike is beyond the scope of this manuscript.

The DLF-Slow operation seems to increase the signal intensity over the entire area for compensating the intensity difference in the low signal intensity section as representatively seen in the line profile after 200 pixels in Fig. 4b. This means that the signal intensity of DLF-Slow processed images would match with that of slow scan images only within a limited signal intensity range. In fact, the PSNR calculated from the DLF-Slow images recorded within a limited signal intensity range of 20,000–40,000 was increased by 13% from the rapid scan images in average. The PSNR calculation outside the signal intensity range (0–20,000 and 40,000–65,535), on the other hand, showed only a 2% increment. The signal intensity matching within a limited range suggests that datasets with various signal intensity ranges should be used in the training process to avoid over-compensation. In this study, the training dataset was acquired in a way that both the minimum and maximum signal intensities fit into a certain range rather than using the entire 16bit dynamic range of the detector, for example, the minimum and the maximum intensities in one area were 7000–10,000 and 17,000–20,000, respectively, while those in another area were 15,000–20,000 and 30,000–40,000. Therefore, to improve the PSNR, it might be needed to acquire images with varying the signal intensity range or non-linearly adjust the contrast such as adaptive histogram equalization before training. Besides the small improvement of the PSNR value, the microstructure features of interest, dislocations in this case, were successfully and sufficiently recovered by the DLF-Slow operation, indicating that the DL-based filter trained by slow scan images showed satisfactory performance.

To further evaluate the image quality, this study discusses also the line profiles of FFT spectrums in a horizontal direction as shown in Fig. 4c, where the natural logarithm of power spectrum (squared amplitude of FFT processed intensity (*F*(*I*)) is evaluated. In this study, all the FFT spectra are obtained after applying the Hanning window to the original images. The line profiles of the DCFI image and the DLF-Slow image demonstrated discrepancy from the low frequency region. The more rapid fall of in the Fourier spectrum along the slow scan direction of the DCFI corrected image relative to the DLF-slow scan corrected and reference images demonstrates the ability of the DLF algorithm to recover spatial information lost in fast scanning. The FFT spectrum of DLF-Slow image, on the other hand, almost coincided with the slow scan image’s one within the wave number of about 120 $\upmu$m^−1^, which is about 8.3 nm in the real space and about 4 pixels in the original image. Since most of the width of dislocation dips in the original images was more than 4 pixels, the DLF-Slow could successfully reproduce the signals with a high spatial resolution compared to the DCFI.

From the above discussion, it was shown that the DL filter supervised by slow scan images could remove not only the statistical noise, but also the unidirectional blurring, which would come from the delay of the detector response and was difficult to remove even by using the well-known noise filter, BM3D. The DLF-Slow obtained in this study could reproduce the signals from poor signal in the rapid scan images. The DL-based noise filter enabled us to acquire STEM images both fast and accurately.

### Application to in-situ observation

It is well known that the performance of DL network depends on the training data. Therefore, sometimes its performance is limited in a specific case. In this section, the DLF-Slow developed in this study is applied to in-situ heating results for further validation of its generality.

We employed a 20%-cold-rolled FCC poly crystal sample of A1050 grade pure aluminum as the in-situ heating specimen. The sample preparation and equipment are described in "Method" section. The sample was placed on a MEMS heater chip, which can instantaneously raise and lower the temperature. Then, we performed continuous acquisition of 2000 frames of 512 × 512 pixels image with the rapid scanning of 100 ns/pixel for 6 series of in-situ heating observation; raising to 90 °C, 1 °C/s temperature rising in the range of 90–150 °C, 150–210 °C, 210–270 °C, 270–330 °C and 330–400 °C. In each experiment, the temperature was kept constant after it reached the designated temperature. The slow scan images of 5 $\upmu$s/pixel were obtained after each experiment for comparison.

**Figure 5:**
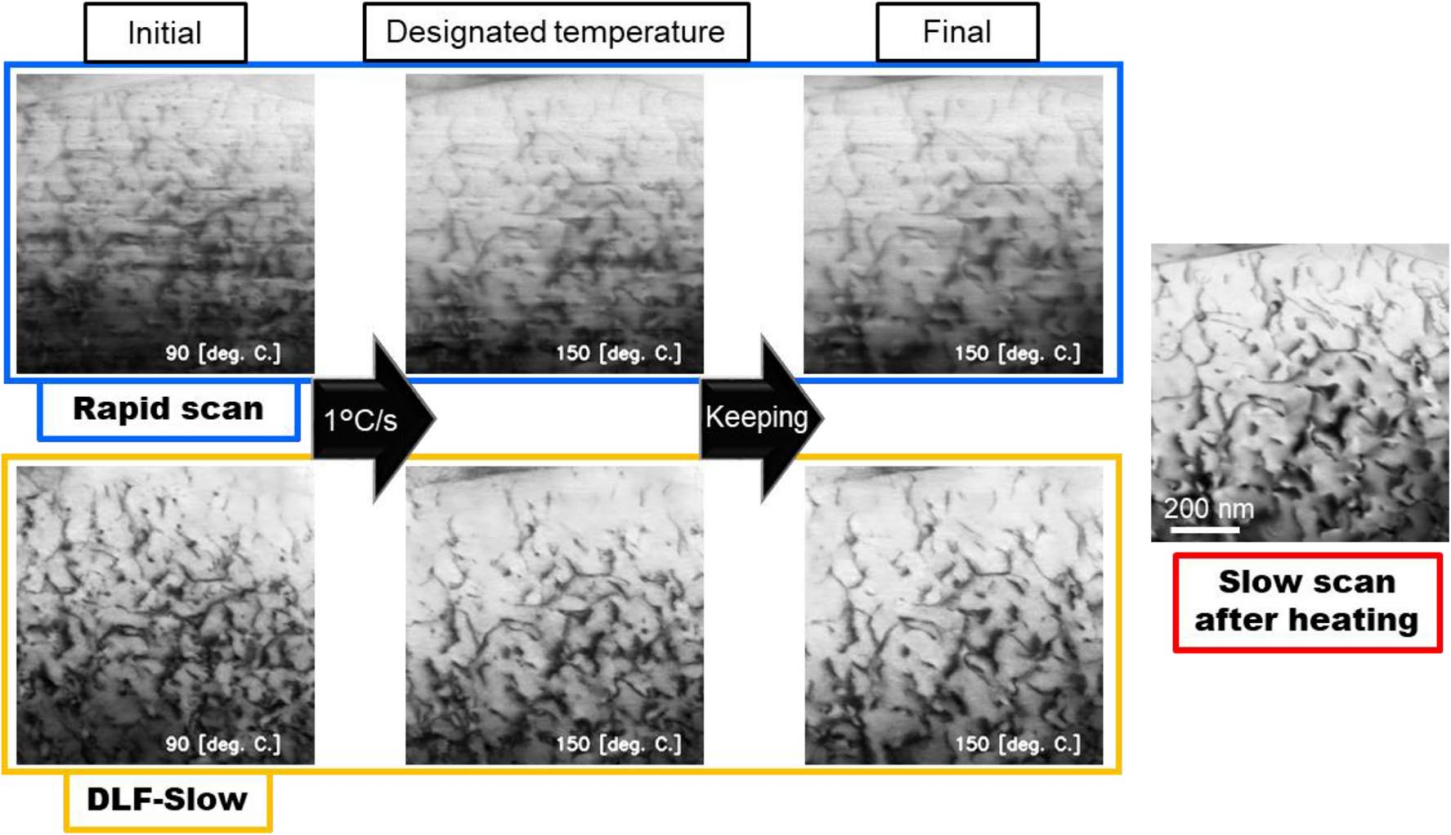
Application of DL-based noise filter to in-situ heating of 20%-cold-rolled pure poly aluminum (A1050). The specimen was heated in a rate of 1 °C/s, then the temperature was kept constant after reaching designated temperature. The heating process from 90 °C to 150 °C is shown here as an example. Other heating processes are represented in Supplementary information C.

Figure 5 shows the results of continuous temperature rising of 90 °C to 150°C as a representative case. The other results are shown in Supplementary information C and those movies are available in Supplementary movie. In Fig. 5, it is clear that the noise contained in the rapid scan images was removed by the DLF-Slow and the dislocations are clearly visible in the DL filtered images. The final state was also similar to that obtained by the slow scan; a 14% increase in PSNR. Therefore, the superior performance of DLF-Slow was also demonstrated for in-situ observation in this temperature range.

### Single FOV training

The DLF-Slow has evidenced high performance for eliminating the statistical noise and the unidirectional image blurring. In this study, the noise filter was made by training either 50 frames × 50 FOVs sets of the rapid scan images or the corresponding view of the slow scan images. Although this dataset was enough for denoising, a possibility that only single FOV might be enough arises if we just desire to remove the statistical noise and the blurring, because these artifacts would be included even in the single FOV. Since vast majority of nanoscale phenomena, e.g., dislocation emission from a crack tip, are site-specific, the observation area is sometimes limited in a few FOVs. The DL noise filter would be required even in in-situ observation that is difficult to obtain the training data. In the following, we examine whether the artifacts can be removed by training with multiple frames taken from only a single FOV or not.

**Figure 6:**
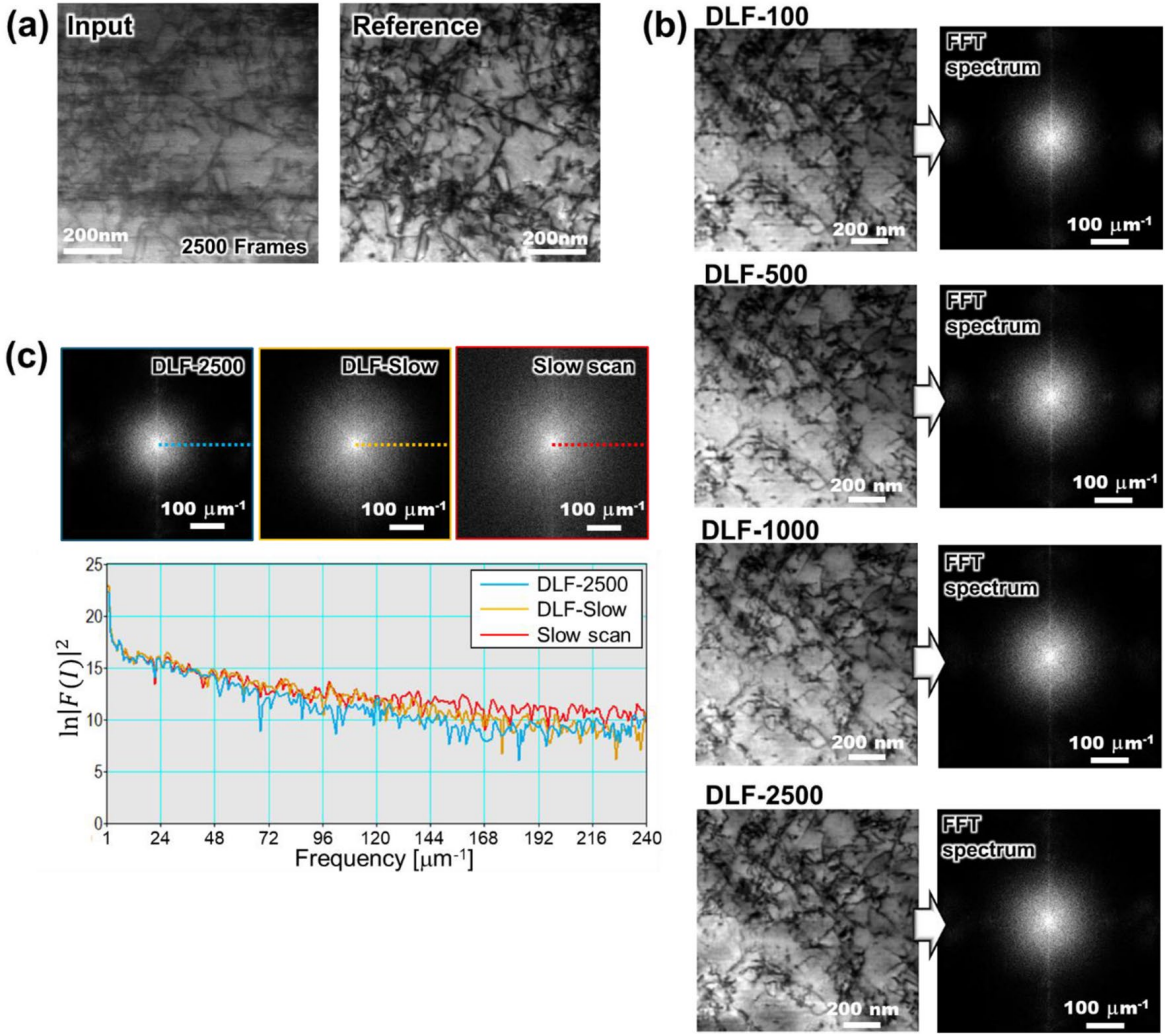
Single FOV training procedure and its results. We performed additional observation and obtained (**a**) 2500 frames of rapid scan images and a slow scan image (reference image) from single view. The data set shown in (**a**) has already processed by the distortion correction. The results of single FOV trained DL filters are shown in (**b**), where DLF-xxx means xxx [frames] of (**a**) were used during the training. (**c**) The line profiles of FFT spectrum are evaluated in the same way as Fig. 4c.

A new set of data including 2500 frames was newly acquired. Figure 6a shows the new distortion-corrected training dataset acquired under the same condition as the previous dataset except for the magnification and the pixel size (here were 99,000 and 1.7 nm/pixel, respectively.). We newly trained the U-net by using up to 2500 frames of the rapid scan images, i.e., 100, 500, 1000 and 2500 frames. In the rest of this paper, each trained DL filter is referred to as DLF-100, DLF-500, DLF-1000, and DLF-2500, respectively. The training conditions were also the same as above section except the learning rate. Since the training data set shown in Fig. 6a contains a lot of dislocations and entangle of those, the U-net risks over-fitting, resulting in a loss of denoising capability overall. To avoid over fitting, we set the learning rate from 0.001 to 1 × 10^−5^ for DLF-100, DLF-500, DLF-1000, or 5 × 10^−6^ for DLF-2500. Figure 6b demonstrates the sample images after applying each filter, where each FFT spectrum is also shown. In the FFT diagrams, the statistical noise was removed even in DLF-100, although the unidirectional blurring was remained. The filtered image became more evident with increasing the number of training data. The vertical spectrum representing the blurring, however, could not be removed even in the DLF-2500.

The line profiles of FFT diagrams are also evaluated as shown in Fig. 6c. The DLF-2500 image is chosen as a representative case of the single FOV training. In Fig. 6c, the line profile in DLF-2500 did not match that of the slow scan after passing the low frequency region of about 48 $\upmu$m^−1^. Since the matching was lower than the DLF-Slow, which almost coincided up to around 120 $\upmu$m^−1^, the ability for reproducing slow scan-equivalent-image-quality became inferior if only the single FOV was used for the training. The reason why the single FOV training was less effective than the multi FOVs training might be because of the dislocation-shape dependence or dislocation-signal-intensity dependence of unidirectional blurring as has been shown in the contour plot of Fig. 1d. During the training process, the DL network is trained so that it reproduces the slow scan images from the corresponding rapid scan images. The transformation function to modify the difference in background intensity would be educated in this process. The background intensity of rapid scan images, however, contains the unidirectional blurring whose shape depends on the shape of dislocations or the intensity of dislocation signal. Therefore, it would be needed to learn multiple patterns of dislocation signal to eliminate the blurring. Although the single FOV training could partially eliminate both the statistical noise and the blurring if the number of frames was increased, the denoising performance would likely be limited because the DL network would not learn the dislocation-shape-dependence or the dislocation-signal-intensity-dependence of the blurring.

In conclusion, the single FOV trained DL filter could remove the image blurring, although the performance was inferior to 50 FOVs trained one. This study indicated that if more FOV were included for training, the performance of DL noise filter would become better. In the case where obtaining many different FOVs are practically challenging, the DL filter will still be worth training to eliminate the statistical noise. This study has also demonstrated that the poor electron signals caused by the rapid scanning in the STEM could be recovered by using DL filtering operations. Therefore, the DL-based technique could be applied mostly in in-situ observation by using the STEM, where temporal resolution is required.

Since the STEM is generally more tolerant of thicker samples, in-situ observation by using the STEM can capture dynamic evolution of phenomena with less effects of surface. The STEM also enables us to obtain the chemical components and bonding state, etc., at the same time when we observe a texture of a sample, providing a large amount of data even in one experiment. Such a big data-like data acquisition could be a standard in the current data-driven materials science. Because of these reasons, the STEM would be a better tool compared to the CTEM for operand observation utilizing data science. Therefore, the denoising technique developed in this study could be applied to determine which area we should further examine by advanced analysis methods, e.g., electron energy loss spectroscopy, as well as to improve the quality and accuracy of STEM images taken with a high temporal resolution. This paper has demonstrated an essential approach for electron microscope observation, which is difficult to establish in the conventional TEM-based way such as observing a dynamic evolution of dislocation structures in a thick sample under external stimuli.

## Method

### STEM image acquisition for training

We employed a FCC single crystal sample of SUS316L grade stainless steel with 3% of compressive pre-strain, which was applied by strain rate of 1 × 10^−4^ s^−1^ along < 1 0 0 > direction, as a specimen. The specimen was ion-polished by Ar^+^, by using a cryo-ion slicer (JEOL Ltd.) to make a foil.

To obtain STEM images, we operated a transmission electron microscope (TEM) Titan Cubed G2 (Thermo Fisher Scientific Inc.) at an acceleration voltage of 300 kV under the STEM mode with a relatively small convergence semi-angle of the incident electron beam, 1.2 mrad. The specimen tilt was carefully adjusted to an exact Bragg condition (so-called two-beam condition^40^) and only the direct beam was detected to obtain high contrast dislocation images. Thus, all the dislocation images were BF images acquired under the condition that only a reciprocal vector of (0 2 2) was excited. A commercial software Velox (Thermo Fisher Scientific Inc.) equipped in the TEM was used to acquire the STEM images. In this study, the STEM images were set to 512 × 512 pixels of 16bit images and taken by the rapid scan at 100 ns/pixel, with continuous acquisition of 50 frames, and the slow scan at 5 $\upmu$s/pixel to a same FOV. Therefore, we obtained 50 frames of the rapid scan image, one 50-frame-averaged DCFI, and one slow scan image for one FOV. All the images were acquired by the magnification of 80,000 with a pixel size of 2.1 nm/pixel. The total was 66 FOVs, where the 50 FOVs were used for the training, and the rest were utilized as test images.

The STEM images used in the single FOV training (Fig. 6a) were acquired by the magnification of 99,000 with a pixel size of 1.7 nm/pixel. Other conditions were the same as above, and continuous acquisition of 2,500 frames of rapid scan images and one frame of slow scan image were obtained. The test images were also the same as the multiple FOVs training.

### Definition of PSNR

The PSNR in this study was defined as,

$$PSNR=10{\mathrm{log}}_{10}\frac{{MAX}_{I}^{2}}{MSE},$$

where $MSE$ is mean squared error between a reference image and a targeted image, and ${MAX}_{I}$ means the maximum pixel value that an image can take. We had acquired the STEM images in 16bit, therefore, ${MAX}_{I}=65535$. Prior to the calculation of PSNR, signal intensity of all images was adjusted by our in-house code so that the minimum and the maximum intensity equal to 0 and 65,535 (the maximum of 16bit image), respectively.

### Distortion correction

**Figure 7:**
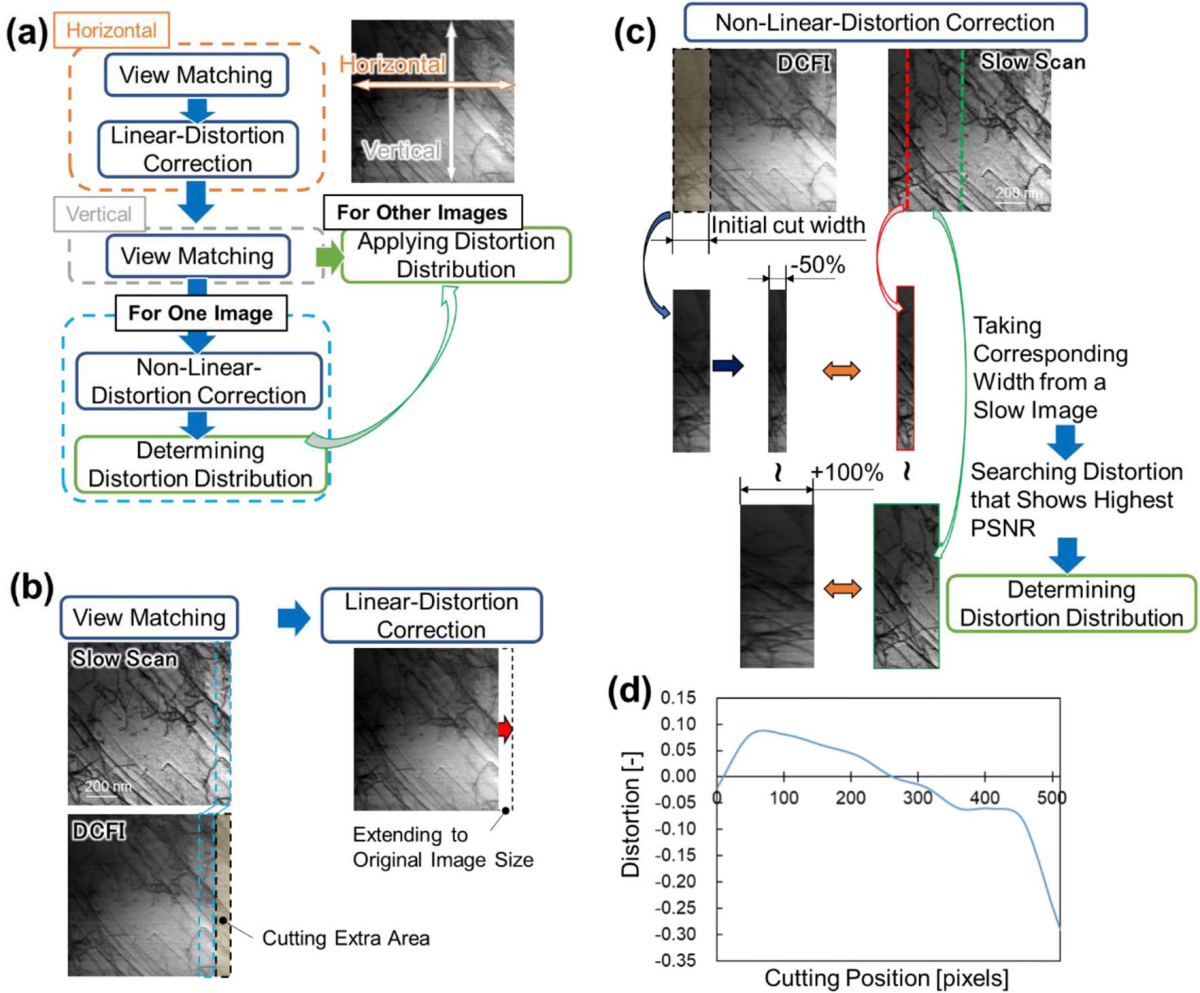
Overview of distortion correction process. This process is roughly divided into three processes, i.e., view matching, linear distortion correction, and non-linear distortion correction, as shown in (**a**) flow chart. (**b**)The view matching and the linear distortion correction are conducted before (**c**) the non-linear distortion correction. The calculated distortion distribution is shown in (**d**).

Figure 7a shows a flow chart of distortion correction process employed in this study. It composed of roughly three processes, i.e., view matching, linear distortion correction, and non-linear distortion correction. Each process consisted of mainly the calculation of cross-correlation to the slow scan images. If there are no dislocations or dislocations lie only on the scanning direction, a deformed rapid scan image is identical to its original image, resulting in failure of distortion correction. The image containing dislocations entirely and randomly was ideal to determine accurate distortion. Thus, for calculating the non-linear distortion correctly, we selected an ideal FOV in which the dislocations were heterogeneously distributed. Then, the obtained distortion was applied for the rest of images. The following describes the details of each method represented in Fig. 7a.

Figure 7b shows the initial processes for the series of distortion correction. Since the rapid scan images contains extra view compared to the slow scan images due to the distortion, we had to firstly modify the view by aiding the cross-correlation. The distortion made it challenging to match the view at once, thus, we divided the process into the horizontal and the vertical direction. We let the template with the width of 50 pixels taken from the edge of the slow scan image be scanning on the corresponding rapid scan image to find the corresponding position showing the highest zero-mean normalized cross-correlation (ZNCC). The outside was removed, leaving the cut image. Then, it was linearly stretched to the original size for the horizontal direction. The process was carried out both left and right side of images. For the vertical direction, on the other hand, the cutting of image was only at top or bottom of image which showed higher ZNCC, and the stretching was not carried out, because the distortion was limited to the horizontal direction, which is the scanning direction of electron beam, in this study. Note that the vertical direction was almost coincide with the slow scan images compared to the horizontal direction in this study. These cuttings and deformation were applied to all the rapid scan and DCFI images. The slow scan images were also to be cut if their view was drifted.

After the view matching, we corrected the non-linear distortion as shown in Fig. 7c. We initially extracted a part of the DCFI image from the left most side, then, let it be deformed from − 50% to 100% in the horizontal direction. The deformed image was to be used to calculate the PSNR to the part of slow scan image taken from the left most side and having the corresponding width. Then, the deformation that showed the highest PSNR was chosen as a distortion of that position. The deformed image portion and the corresponding rectangle taken from the slow scan image were removed for the rest of the process. It means that the non-linear distortion correction for the next was carried out from the left most side of the rest of uncorrected image. The same procedure was applied until the initially cut rectangle reached the right side of image. To avoid the dependency of the initial cut width, we set the width from 10 to 100 pixels and chose the width scoring the highest PSNR at the finally obtained image. Figure 7d shows the distortion distribution obtained in this study, where plotting the cutting position and its distortion calculated in the above process. In this study, the distortion between the cutting position was interpolated by cubic spline. This distortion distribution was applied to all the rapid scan images. The representative result of distortion correction has been shown in Fig. 1c.

### Deep learning

**Figure 8:**
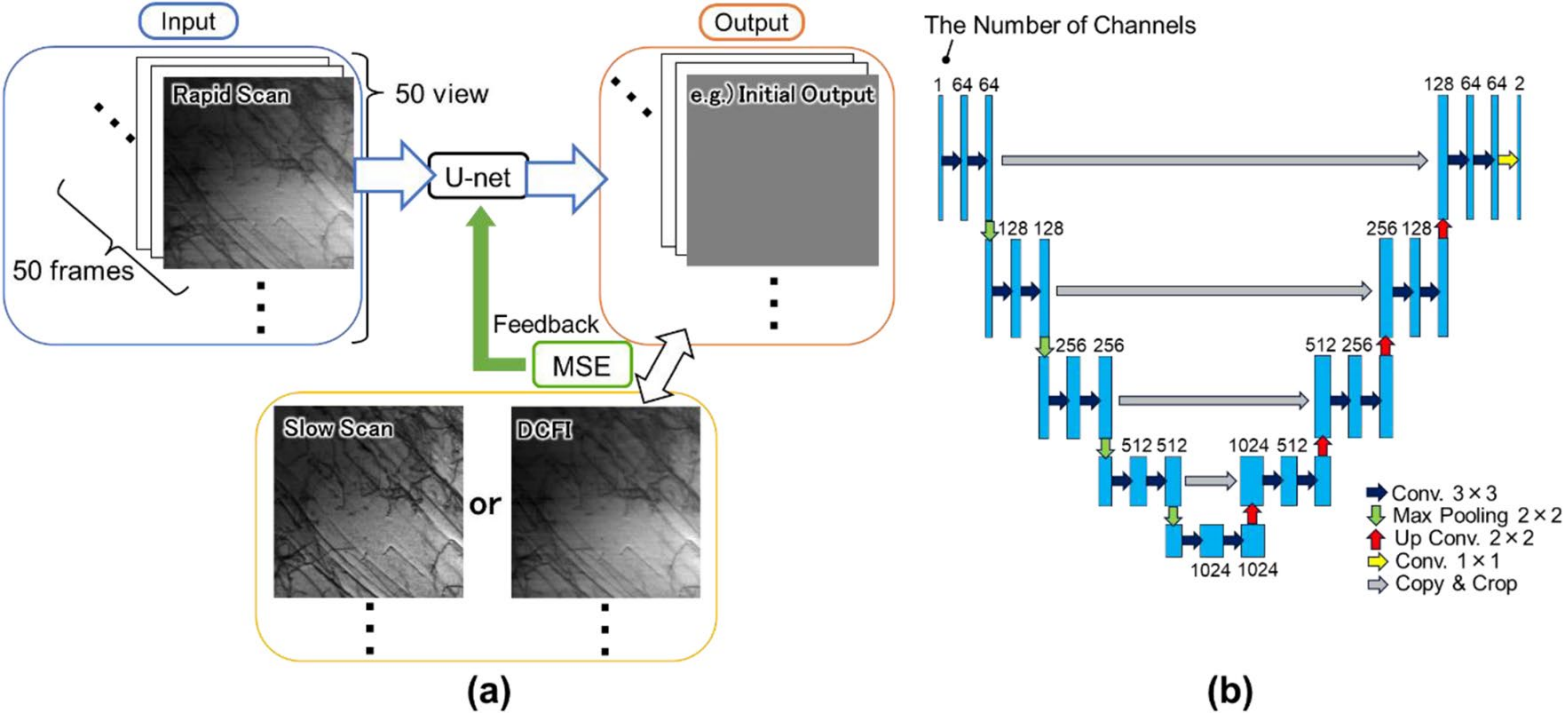
Summary of DL process. (**a**) Schematic drawing of DL procedure in this study. The architecture of DL algorithm is shown in (**b**)^39^.

Figure 8a shows the DL scheme employed in this study. As noted in “STEM Image Acquisition”, we prepared 50 frames of the rapid scan image per one FOV, and total of 50 FOVs for training. 20% of image data set were utilized as a validation data during the training. The slow scan images and the DCFI images were duplicated to correspond with the number of the rapid scan images, and set to be the reference. Note that all the training images were already corrected by the distortion correction as described in Fig. 7a, and their brightness was adjusted so that the minimum and the maximum intensity equal to 0 and 65,535 (the maximum of 16bit image), respectively. The DL architecture utilized here was U-net^39^, well known as a U-shaped network as shown in Fig. 8b, which is implemented in a commercial software, Dragonfly (Object Research Systems Inc.)^41^. We trained the network with the following condition; patch size was 64 × 64 pixels, loss function was Mean Squared Error (MSE), optimization algorithm was Adam^42^, learning rate was 0.001, and total epoch was 100. The single-view training, on the other hand, the learning rate was decreased to 1 × 10^−5^ for DLF-100, DLF-500, and DLF-1000, and 5 × 10^−6^ for DLF-2500. Other conditions were identical.

### In-situ observation

As noted in the Application to in-situ observation, a 20%-cold-rolled FCC poly crystal sample of A1050 grade pure aluminum was employed. The specimen was thinned by using focus ion beam (Versa3D, Thermo Fisher Scientific Inc.) and the foil was placed on a MEMS chip (Norcada), which is composed of a SiN thin film with an electrode lithography pattern. Double Tilt 4 Electrodes Transfer Holder (Mel-Build) was used for the in-situ heating. Image acquisition condition was same as the condition described in the STEM image acquisition for training section except the magnification and the pixel size (here were 99,000 and 1.7 nm/pixel, respectively).

## Supplementary Information

Supplementary Information.

Supplementary Video 1.

Supplementary Video 2.

Supplementary Video 3.

Supplementary Video 4.

Supplementary Video 5.

Supplementary Video 6.

Supplementary Video 7.

Supplementary Video 8.

Supplementary Video 9.

Supplementary Video 10.

Supplementary Video 11.

Supplementary Video 12.

## Data Availability

The codes employed for the noise filtering and distortion correction are available from the corresponding author upon reasonable request.
