# Supplementary material for: Deep learning-based noise filtering toward millisecond order imaging by using scanning transmission electron microscopy
Source: Sci Rep. 2022 Aug 5;12:13462. doi: 10.1038/s41598-022-17360-3 (PMC9356044; doi:10.1038/s41598-022-17360-3)
Supplement: Supplementary file 1 — Supplementary Information. [file 41598_2022_17360_MOESM1_ESM.docx]

*Supplementary Information*

Deep learning-based noise filtering toward millisecond order imaging by using scanning transmission electron microscopy

*Shiro Ihara*^1*^, *Hikaru Saito*^1,3^, *Mizumo Yoshinaga*^2^, *Lavakumar Avala*^1^, and *Mitsuhiro Murayama*^1,4,5^

^1^ Institute for Materials Chemistry and Engineering, Kyushu University, Fukuoka 816-8580, Japan

^2^ Interdisciplinary Graduate School of Engineering Sciences, Kyushu University, Fukuoka 816-8580, Japan

^3^ Pan-Omics Data-Driven Research Innovation Center, Kyushu University, Fukuoka 816-8580, Japan

^4^ Department of Materials Science and Engineering, Virginia Tech, Blacksburg, VA 24061, USA

^5^ Reactor Materials and Mechanical Design Group, Energy and Environmental Directorate, Pacific Northwest National Laboratory, WA 99352, USA

* ihara-shiro@cm.kyushu-u.ac.jp

1. **Scan distortion**

As discussed in the main article, the rapid scanning of 100 [ns/pixel] causes the image distortion and we have proposed the distortion correction method by using the slow scan image acquired in 5 [μs/pixel] as a reference. Although the distortion correction has shown the successful performance, it should be confirmed that the slow scan images are not distorted. In this section, we examine the image distortion in the slow scan images by using a TEM standard sample, MAG*I*CAL (Ted Pella, Inc.).

We acquired 512×512 [pixels] of high-angle-annular dark field (HAADF) images of the standard sample. Figures A.1(a) and (b) show the HAADF images, where the magnification and the pixel size in the former are 70,000 and 1.7 [nm/pixel], respectively, while those in the latter are 99,000 and 2.3 [nm/pixel], respectively. The red line in the slow scan images show the top of the band, while those in the rapid scan images were drawn so that they were parallel to the red line in the corresponding slow scan image and starting from the upper right corner. In Figs.A.1(a) and (b), it is clearly demonstrated that the slow scan images represented no image distortion as the measured width was same in both right and left side, meaning the lines in the images are parallel to each other. We can also confirm that the rapid scan images showed the image distortion because the distance between the lower right corner and the upper right corner differs from that between the lower left corner and the left edge of the red line. It should be noted that the HAADF images and the bright field (BF) images were coincided each other as shown in Fig.A.1(c). Therefore, the results demonstrated in Figs.A.1(a) and (b) are also applicable to BF images, and the slow scan images used in the distortion correction ware not distorted.

| 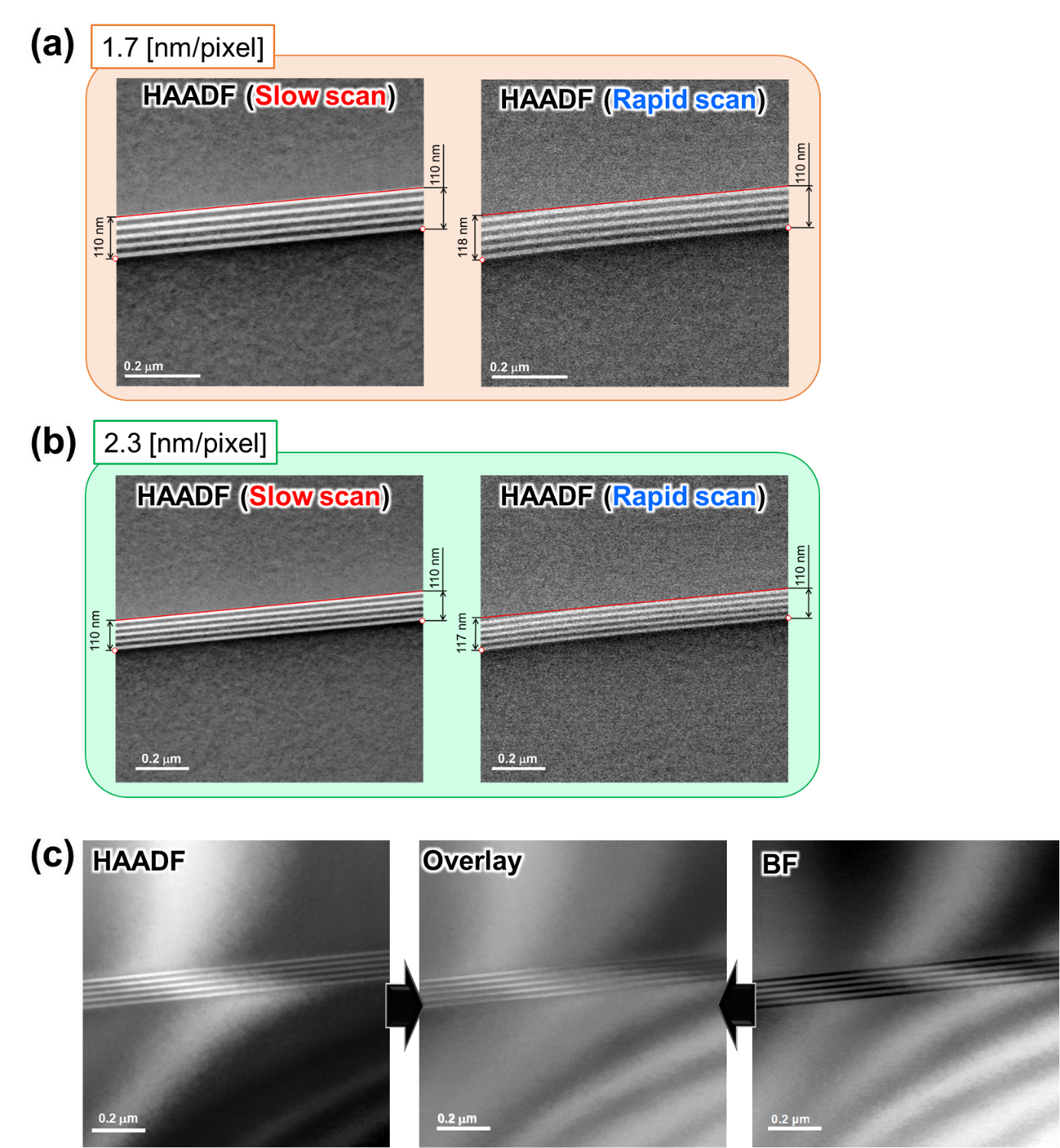 |
| --- |
| Fig.A.1 Measurement of image distortion for rapid (100 [ns/pixel]) and slow scan (5 [μs/pixel]). No distortion was confirmed in slow scan as the height was not changed between the left edge and the right edge. |

1. **Results of deep learning filter with and without distortion correction for entire field-of-view**

This section shows results of deep learning filter (DLF) trained with and without the distortion correction (DC) described in the Method section. The test images of 50[frames]×16[field-of-view (FOVs)] were processed through the DLF. Figure B.1 indicates of the rapid scan images and those filtered by DLF without DC and DLF with DC, respectively, for all fields-of-view, where the slow scan images are also shown as a reference. The DLF with DC is the same as DLF-Slow in the main article and the DLF without DC was trained in the same condition as the DLF-Slow. As noted in the main article, the rapid image acquisition of STEM causes non-linear image distortion as clearly visualized in Fig.1(a), resulting in the misfit of dislocation position. Without the DC, the DLF generated undesired artifacts as shown in the second column of Fig.B.1, because the DL network would try to fit each of dislocation positions, whose process involves both the elimination of dislocations in the original images and the generation of those at positions where dislocations exist in the reference images. The transformation which requires the generation of objects risks artifact generation and might need numerous training dataset to prevent it. The DLF with DC, on the other hand, successfully reproduces the slow scan images for all fields-of-view. Therefore, the effectiveness of the DC has been clearly represented in the figure.

| 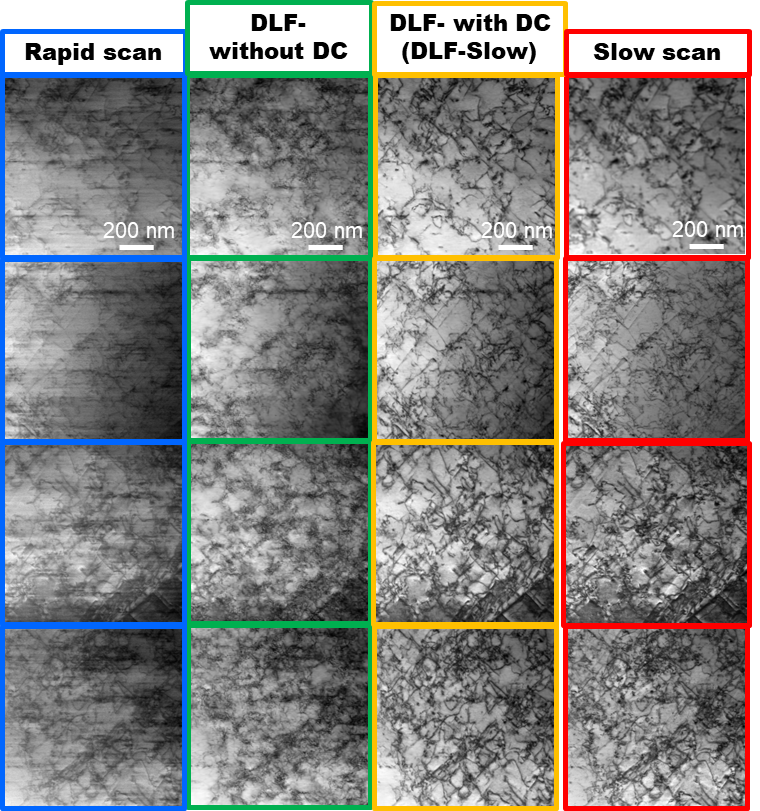 |
| --- |
| Fig.B.1 Rapid scan images and results of DLF processed images for all FOVs. Slow scan images are also shown as a reference. |

| 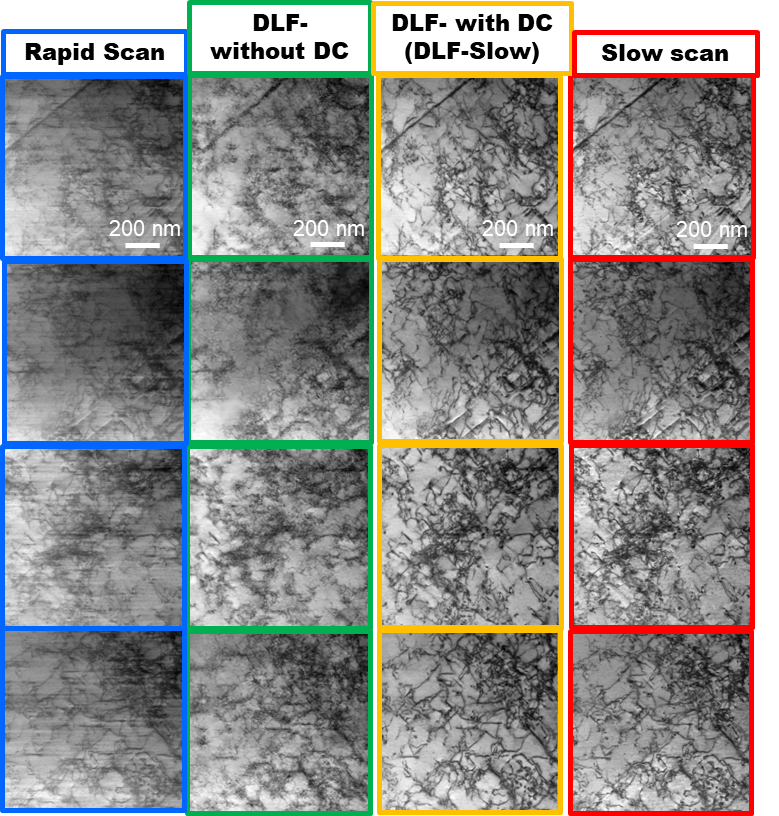 |
| --- |
| Fig.B.1 Continued |

| 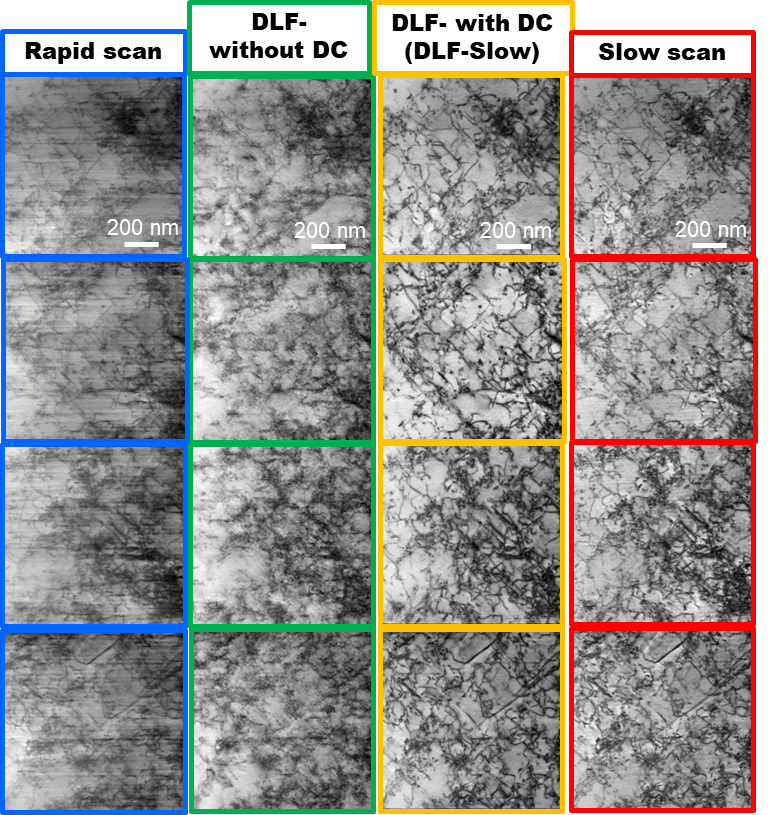 |
| --- |
| Fig.B.1 Continued |

| 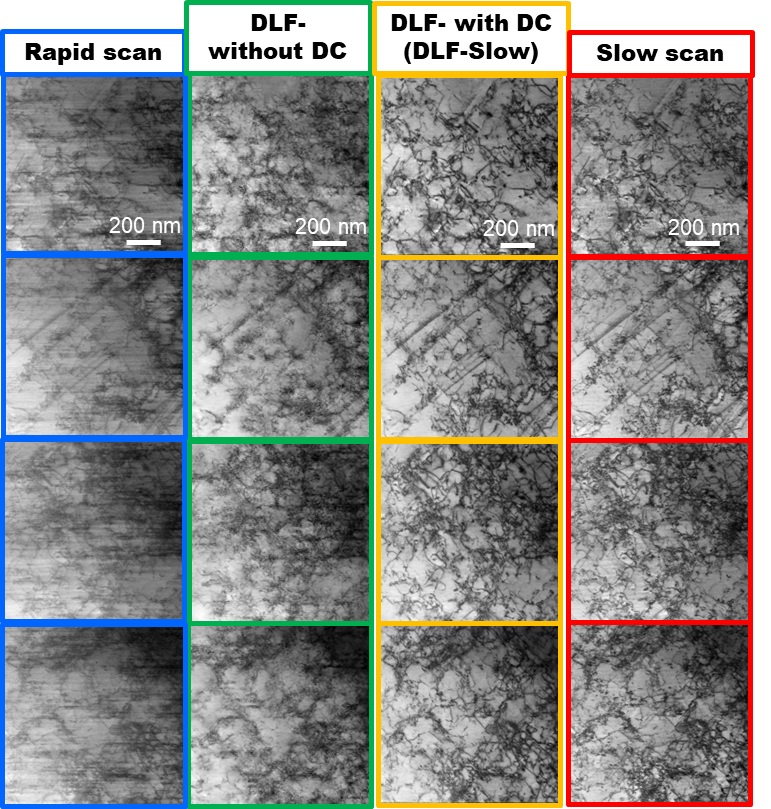 |
| --- |
| Fig.B.1 Continued |

1. **Application of DL-based noise filter to in-situ heating observation**

We performed series an in-situ heating experiment of 20%-cold-rolled A1050 and applied the DLF-Slow. The detail of sample preparation is shown in Method section of the main article. The acquisition time was same as the training data (100[ns/pixel]) and continuous acquisition of 2000 [frames] was performed. In this study, 6 heating conditions were set; keeping temperature after setting 90°C, and continuous temperature rising of 90°C~150°C, 150°C~210°C, 210°C~270°C, 270°C~330°C and 330°C~400°C with a rate of 1[°C /s]. The temperature was kept constant when reaching the designated temperature. After each heating process, the slow scan image (5[μs/pixel]) was acquired to compare a DLF-Slow image.

Figure C.1 shows the results. To see the whole process, see Supplementary movies. In each heating step, the DLF-Slow removed the noise and we can clearly identify dislocations in the processed images. The final states were almost similar to the slow scan images except images, which contain faint dislocation. Particularly, in the case of 210°C-270°C, the dislocations were gradually disappeared and bend contour changed during the heating process because of grain rotation accompanied with recovery process. Since it is difficult to identify the dislocations in that case, the DLF-Slow was failed to recover the signal. To recover the faint dislocations, just increase of dataset or including dataset containing faint dislocations are possible approach.

| 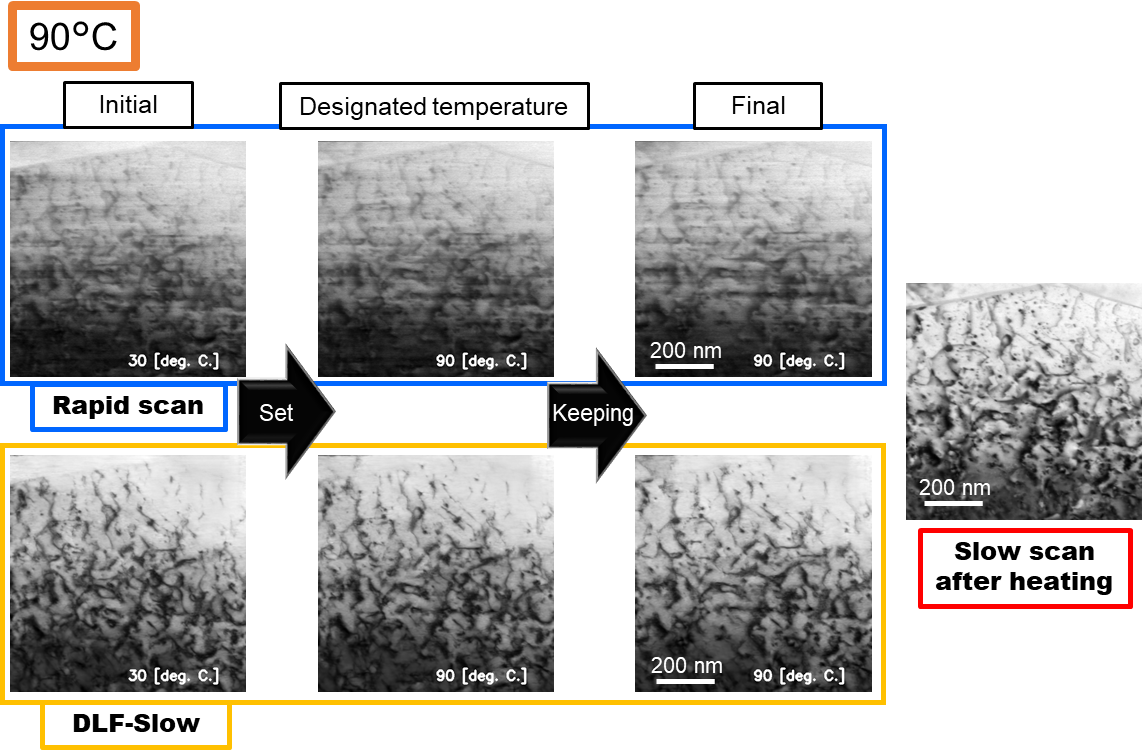 |
| --- |
|  |
| 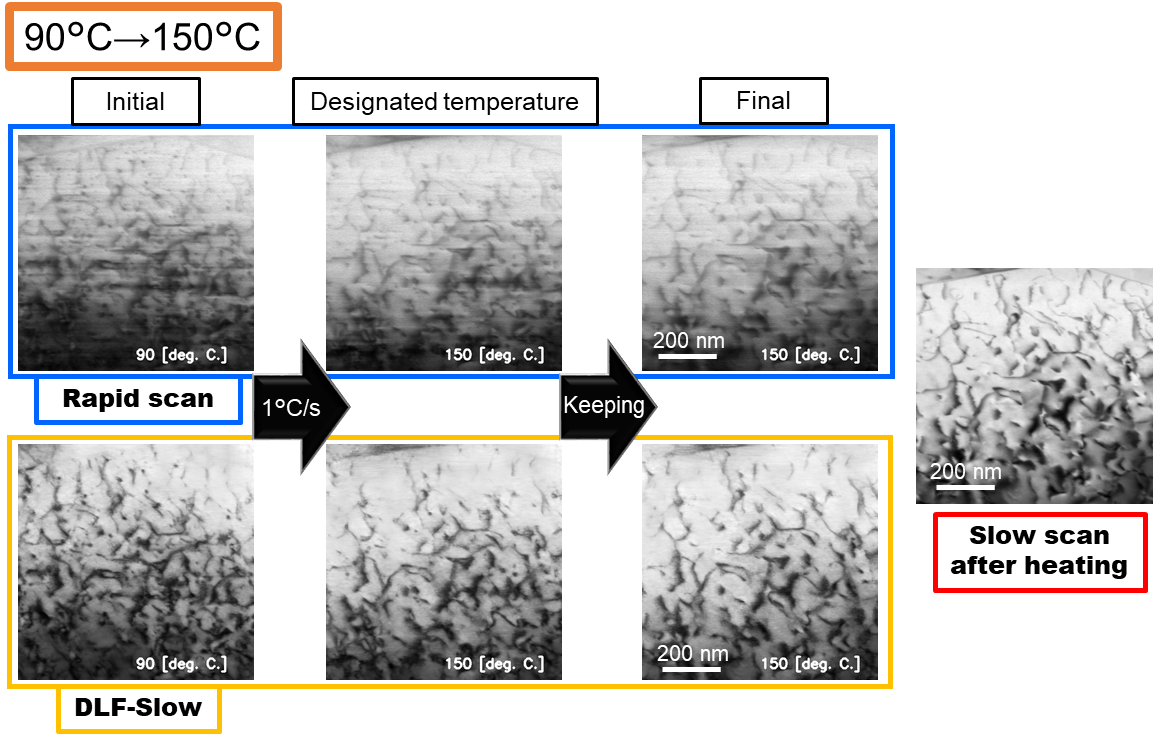 |
| Fig.C.1 Results of in-situ heating of 20%-cold-rolled A1050. The rapid scan images were denoised by DLF-Slow. |

| 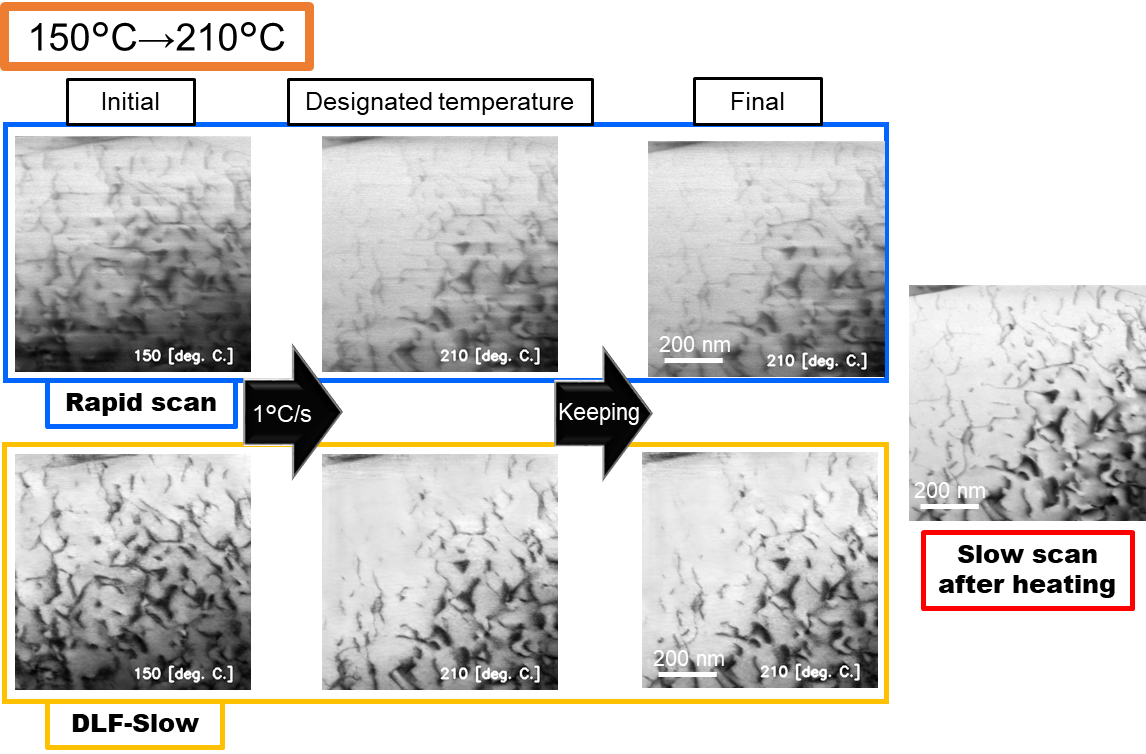 |
| --- |
|  |
| 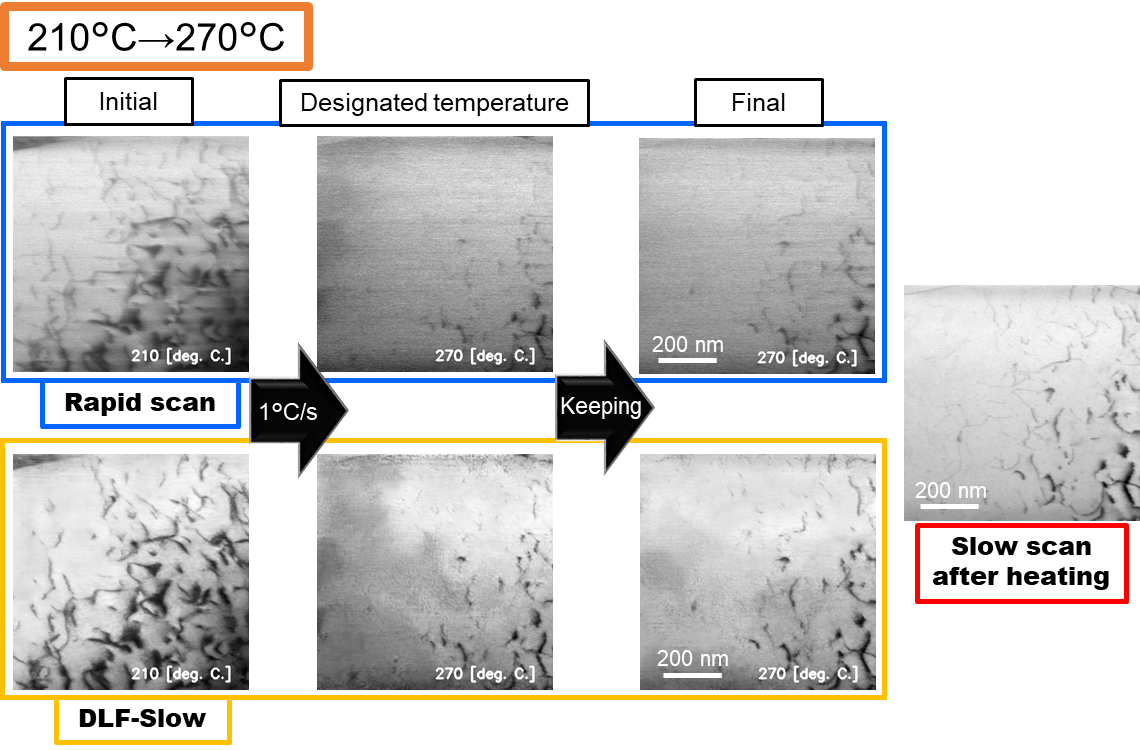 |
| Fig.C.1 Continued |

| 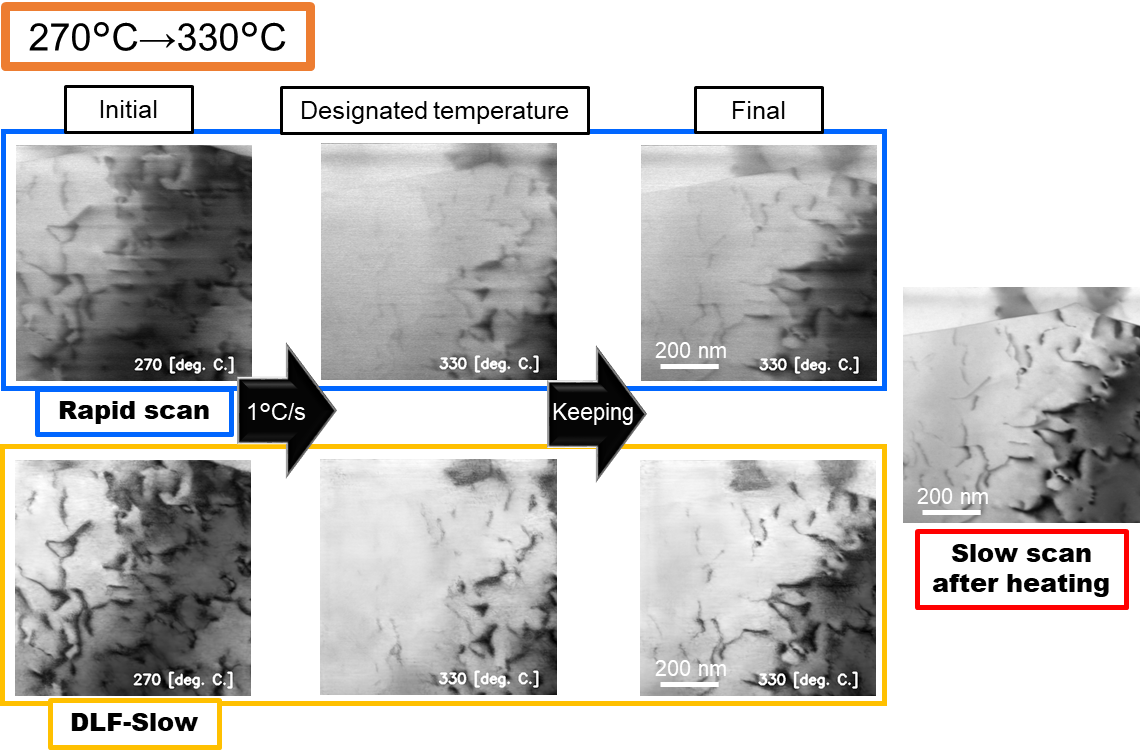 |
| --- |
|  |
| 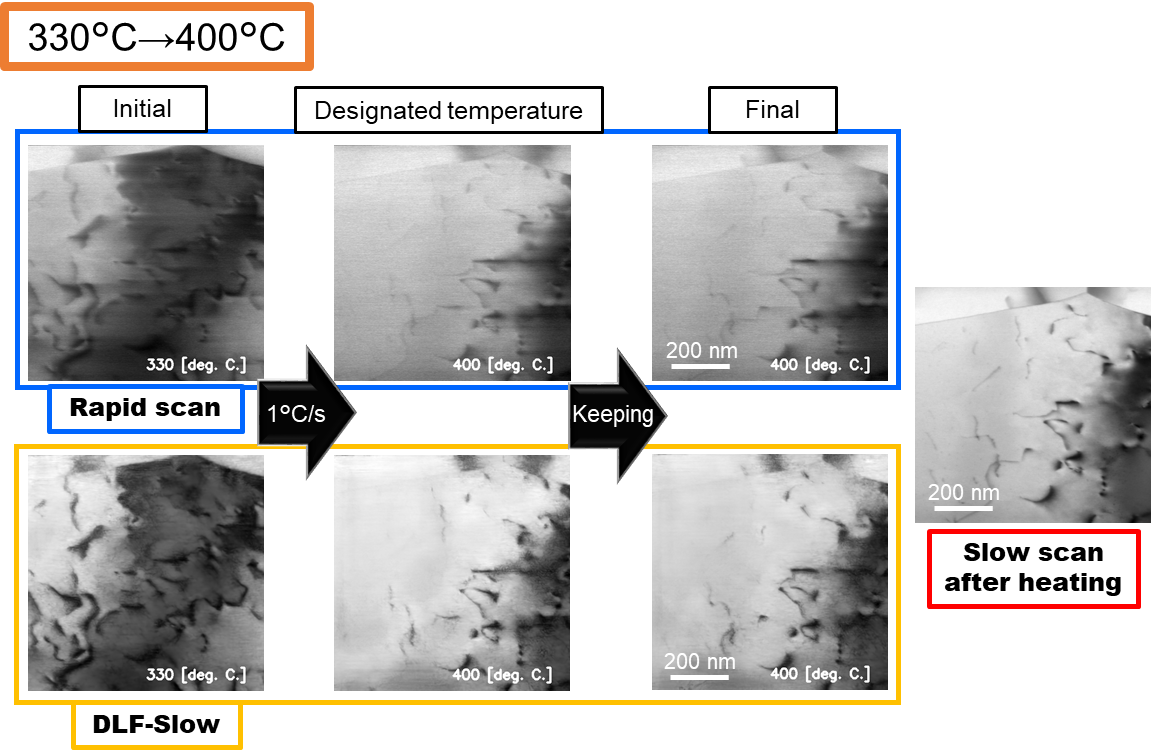 |
| Fig.C.1 Continued |
